# Discrepant outcomes in two Brazilian patients with Bloom syndrome and Wilms’ tumor: two case reports

**DOI:** 10.1186/1752-1947-7-284

**Published:** 2013-12-30

**Authors:** Marilia Borges Moreira, Caio Robledo DC Quaio, Aline Cristina Zandoná-Teixeira, Gil Monteiro Novo-Filho, Evelin Aline Zanardo, Leslie Domenici Kulikowski, Chong Ae Kim

**Affiliations:** 1 Genetics Unit, Instituto da Criança do Hospital das Clínicas da Faculdade de Medicina da Universidade de São Paulo, São Paulo, SP, Brazil; 2 Department of Pathology, Cytogenomic Laboratory – LIM03 – Hospital das Clínicas da Faculdade de Medicina da Universidade de São Paulo, São Paulo, SP, Brazil; 3 Unidade de Genética do Instituto da Criança, HC – FMUSP, Av. Dr. Enéas Carvalho de Aguiar, 647, CEP: 05403-000, São Paulo, SP, Brazil

**Keywords:** Bloom syndrome, Cancer risk, Chromosomal instability, Sister chromatid exchange, Wilms’ tumor

## Abstract

**Introduction:**

Bloom syndrome is a rare, autosomal recessive, chromosomal instability disorder caused by mutations in the *BLM* gene that increase the risk of developing neoplasias, particularly lymphomas and leukemias, at an early age.

**Case presentation:**

Case 1 was a 10-year-old Brazilian girl, the third child of a non-consanguineous non-Jewish family, who was born at 36 weeks of gestation and presented with severe intrauterine growth restriction. She had Bloom syndrome and was diagnosed with a unilateral Wilms’ tumor at the age of 3.5 years. She responded well to oncological treatment and has remained disease-free for the last 17 years. Case 2 was a 2-year-old Brazilian girl born to non-Jewish first-degree cousins. Her gestation was marked by intrauterine growth restriction. She had Bloom syndrome; a unilateral stage II Wilms’ tumor was diagnosed at the age of 4 years after the evaluation of a sudden onset abdominal mass. Surgical removal, neoadjuvant chemotherapy and radiotherapy were not sufficient to control the neoplasia. The tumor recurred after 8 months and she died from clinical complications.

**Conclusion:**

Our study reports the importance of rapid diagnostics and clinical follow-up of these patients.

## Introduction

Bloom syndrome (BS; Online Mendelian Inheritance in Man database, number 210900) [1] is a rare, autosomal recessive, chromosomal instability disorder [2] caused by mutations in the *BLM* gene, which encodes a product necessary for the maintenance of genomic stability [3]. The prominent clinical features associated with BS include severe growth deficiency (pre- and postnatal), sun-sensitive facial erythema, immunological deficiency and a remarkably increased risk of developing neoplasias of various types at a younger age than expected in the general population; the neoplasias are the main cause of death among affected individuals [4,5].

Among the 265 cases of BS reported in the Bloom’s Syndrome Registry (which includes 222 families), 122 developed some type of neoplasia during their lives; leukemias, lymphomas and carcinomas were common, but several other cancers have been reported [4,5]. These patients all present a remarkably increased frequency of sister chromatid exchange (SCE). The cancer predisposition in patients with BS can be attributed to excessive chromosomal breakage and homologous recombination events that lead to spontaneous mutations in the somatic cells and defective damage response functionality [6].

Wilms’ tumor (WT) is the most common pediatric solid cancer; it has been estimated to occur in 1:10,000 children below the age of 15 years and was once considered a rare event among patients with BS [7]. Nevertheless, six patients with BS who developed this tumor have been described previously in the literature [8-11].

Here we report the cases of two new unrelated Brazilian patients diagnosed with BS who developed WT.

## Case presentation

### Case 1

A 10-year-old Brazilian girl, the third child of a non-consanguineous, non-Jewish family, was born at 36 weeks of gestation and was marked by severe intrauterine growth restriction. After resolving the clinical complications of her extreme low birth weight (BW) of 1500g, she was discharged from the hospital; however, despite presenting normal cognitive development during the first year of her life, a remarkable, refractory failure to thrive was noted. She had recurrent diarrhea and upper and lower respiratory tract infections by the age of 1 year and required prophylactic antibiotics. A WT was diagnosed in her left kidney after evaluating an abdominal mass at the age of 3.5 years. She underwent surgical removal of the mass and neoadjuvant chemotherapy (unknown regimen).

The first genetic evaluation occurred at age 10 when a physical examination revealed features characteristic of BS, including facial features (elongated face, prominent nose, prominent ears, malar hypoplasia, and thin upper vermilion), microcephaly, nose telangiectasias, hypomelanotic macules in her upper limbs and clinodactyly of her bilateral fifth fingers. Her anthropometric measurements were all below the fifth percentile (weight (W) 15.9kg, height (H) 116cm, and occipital-frontal circumference (OFC) 46cm; Centers for Disease Control and Prevention growth charts, National Center for Health Statistics, USA), and she had developed learning disabilities.

**Figure 1:**
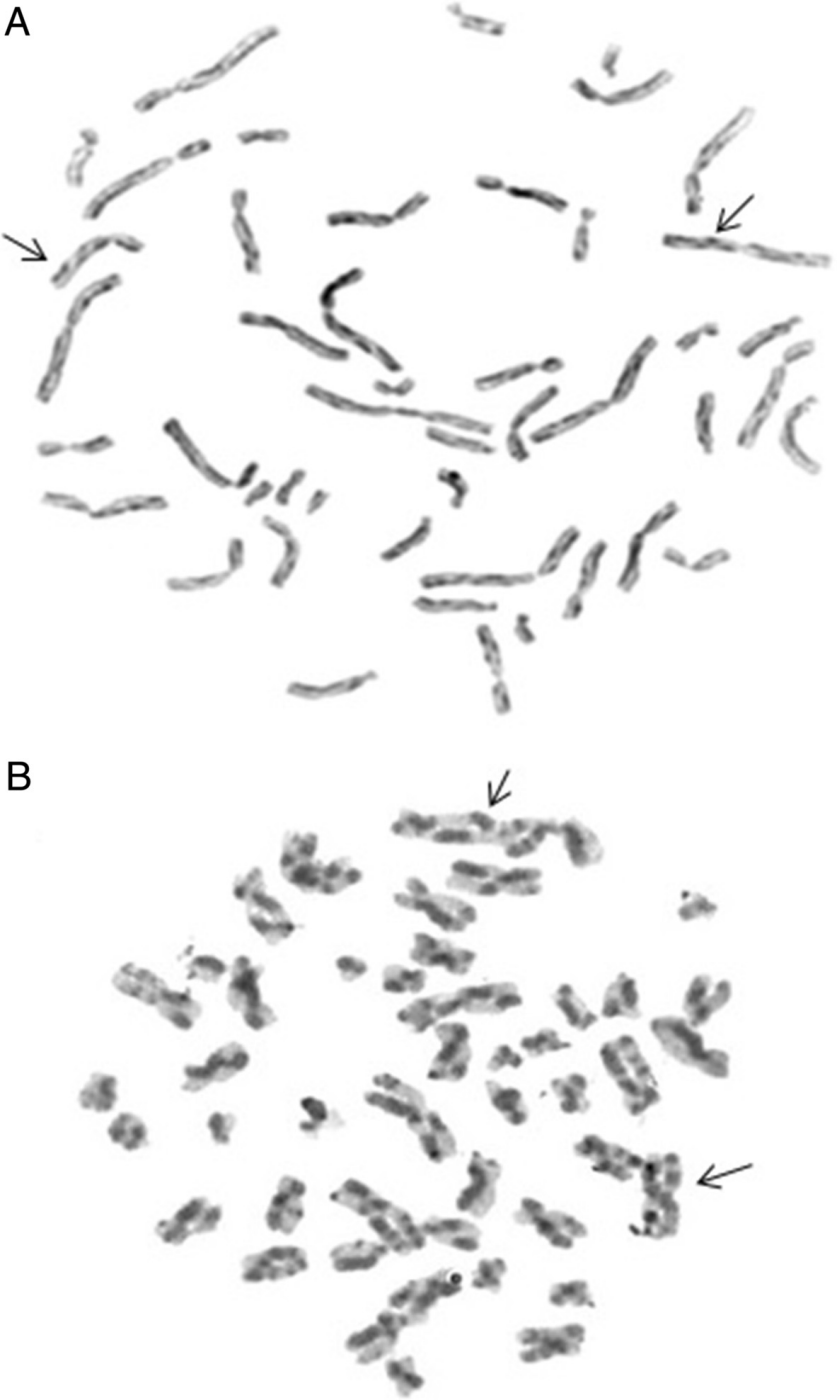
**The picture demonstrates the manifold increase of sister chromatid exchange in Case 1 (A) and Case 2 (B).** The arrows exemplify some points of sister chromatid exchange. The lymphocyte cultures were treated with bromodeoxyuridine and staining with Hoechst 33258-Giemsa.

A cytogenetic test was performed to examine the frequency of SCE using bromodeoxyuridine (BrdU) in lymphocyte cultures. The results demonstrated an increased frequency of SCE, with an average of 48.5 SCE per cell (Figure 1A). The G-band karyotype was normal (46, XX).

She responded well to oncological treatment and has remained disease-free for the last 17 years. Currently, at the age of 21, all of her anthropometric measurements continue to be below the standards for the normal population (H: 140cm; W: 26.4kg; and OFC: 47cm).

### Case 2

The gestation of a 2-year-old Brazilian girl, who was born to non-Jewish first-degree cousins, was marked by intrauterine growth restriction. Vaginal delivery occurred at term with no complications. The neonate had a low BW of 2060g, microcephaly (OFC: 30.5cm) and a normal length (45.5cm); her anthropometric measurements were all below the fifth percentile. A physical examination at the age of 2 years revealed a short stature (78cm), low W (8.1kg), and microcephaly (OFC: 45cm); her anthropometric measurements were all below the fifth percentile. Features characteristic of BS, including facial features (long, narrow face; telangiectasic erythema involving her nose, malar and oral regions; prominent nose; and retrognathia), café-au-lait macules throughout her body, diffuse hypomelanotic macules, and bilateral fifth finger clinodactyly, were identified. Recurrent upper respiratory tract infections were common, but she had not experienced any serious infection or required prophylactic antibiotic use.

A stage II unilateral WT was diagnosed at the age of 4 years after the evaluation of a sudden onset abdominal mass. Surgical removal, neoadjuvant chemotherapy and radiotherapy were not sufficient to control the neoplasia. The tumor recurred after 8 months, and the patient died from clinical complications.

A cytogenetic test was performed to examine the frequency of SCE using BrdU in lymphocyte cultures. The results demonstrated an increased frequency of SCE, with an average of 49.5 SCE per cell (Figure 1B). The G-band karyotype was normal (46, XX).

## Discussion

We described two Brazilian patients affected by BS who each developed a WT at a young age. These two cases, along with the other six described in the literature (Table 1) [8-11], increase the estimated frequency of WT in individuals with BS to at least 3%, a 300-fold increase in the risk relative to the general pediatric population.

**Table 1:** Patients affected by Bloom syndrome associated with Wilms’ tumor

	**Ashkenazim**	**Consanguinity**	**Age at diagnosis of WT**	**References**
1	No	No	3.5y	Our patient
2	No	Yes	4y	Our patient
3	Yes	No	8y	[8]
4	No	No	5m	[8]
5	No	No	22m	[8]
6	No	No	4y	[9]
7	Not mentioned	Not mentioned	3y	[10]
8	No	No	3.5y	[11]

*Abbreviations*: *m* months, *WT* Wilms’ tumor, *y* years.

BS is caused by mutations in the *BLM* gene, whose product is a 1417-amino acid protein that belongs to the RecQL helicase group and plays important roles in replication, recombination and cellular repair. Upon alteration, *BLM* loses its function and causes genomic instability and an increased rate of spontaneous mutations in somatic cells, primarily by SCE [3]. Cytogenetic analyses of cases 1 and 2 have demonstrated an increase in the frequency of exchange between sister chromatids, which is pathognomonic for BS and confirmed the diagnosis of BS [12]. Unequal crossing-over has probably played a major role in the evolution of various genes and heterochromatin. Hypermutability and the hyperrecombinability of somatic cells leads to an increased chance of homozygous tumor suppressor genes and/or oncogenes being affected and, consequently, increases the rate of the development of a wide variety of neoplasias at an early age [5,8]. In some cases, retinoblastoma and WT are associated with the homozygosity of a chromosome segment resulting from a mitotic crossover. Similarly, the high incidence of cancer in BS may be caused by a mitotic crossover that leads to homozygosity or the amplification of oncogenes.

The literature [13] describes that two events lead to the formation of WT. First, the child inherits one genomic alteration, and a second event occurs in the child that would be caused easily in BS because of the high rate of genomic exchanges. Nevertheless, if recombination in BS occurs as a defect in the repair machinery, maybe there is another mechanism that may contribute to the formation of tumors and cancer.

New technological advances in array-based genomics revealed the contribution of structural alterations in the human genome to several different diseases, including cancer (both solid and hematologic tumors) [14]. In fact, certain copy number variations (CNVs) potentially compromise fundamental processes controlling genomic stability, including deoxyribonucleic acid (DNA) replication and the DNA damage response, and have been reported to be associated with the response to chemotherapy, which affects the disease prognosis [12]. From a clinical perspective, CNVs might interfere in the DNA damage response and create a permissive environment for the acquisition of additional pathogenic alterations, such as an individual’s predisposition to cancer [15].

Furthermore, recently, Stephens and colleagues recently described a novel mechanism of genomic rearrangement in cancer cells, termed ‘chromothripsis’, that associates specific CNVs and their contribution to cancer development. Chromothripsis arises through chromosome breakage and inaccurate reassembly, produces highly complex derivative chromosomes, and causes oncogene amplification. A disaster of this magnitude could possibly affect both WT alleles because patients with BS demonstrate susceptibility for breaks in the genome [16].

In our study, both patients developed WT before 4 years of age; however, despite both patients undergoing surgical removal of the mass and chemotherapy, the treatment results were different. The patient described in case 2 (stage II WT) had a fatal outcome after the recurrence of the disease, but the patient described in case 1 has remained disease-free after 17 years of follow-up. All reported cases of WT in patients with BS in the literature and the two cases described here occurred in patients aged 8 years or less.

WT has been previously reported in six cases of BS in the literature [8-11]. The age at which WT was diagnosed ranged from 5 months to 8 years. Two of these cases were diagnosed at early stages (stages I and II) and had apparently been cured by nephrectomy and chemotherapy. Although patients with BS develop cancer at early ages, the age of onset of WT is the same in literature reports of patients without previous medical histories who are under the age of 15 years and in patients with BS [17].

Thus, the frequency of WT in patients with BS is considerable. Clinicians need to be more aware of this fact, particularly because the occurrence of WT in patients with BS was once presumed to be rare. This frequency summed with the frequencies of other solid tumors in patients with BS increases the overall frequency of solid tumors to approximately 12% [5]. Unfortunately, insufficient attention is given to this group of tumors in patients with BS.

Beckwith–Wiedemann syndrome is a prototype genetic disease with an increased risk of the development of early onset solid cancers, mainly WT and hepatoblastoma, with a total estimated lifelong risk for solid tumors of 7.5% [18]. This increased risk has prompted specialists to seriously consider solid tumor surveillance because surveillance has been demonstrated to reduce treatment-related morbidity. Renal ultrasonography is currently the optimal surveillance modality and is accessible, is non-invasive and has minor risks [19]. However, this method may have unfavorable consequences because false positive results may lead to unnecessary investigations and surgical procedures.

Although the evidence does not show benefits for leukemia screening in patients with BS because early treatment does not improve clinical outcomes, surveillance for solid tumors may have significant advantages and improve survival [9,10]. In addition, the frequency of solid tumors in patients with BS is approximately the same as that for patients with Beckwith–Wiedemann syndrome, and a screening program has been demonstrated to be feasible in the latter patients. Thus, we suggest that ultrasound be performed as a regular method of surveillance for the early detection of solid tumors in individuals with BS because the early detection of these tumors may have clinical benefits. The frequency of this type of screening must be individualized, but the performance of an ultrasound examination at least every 6 months is advisable based on the vast experience with Beckwith–Wiedemann syndrome reported in the literature.

Cytogenetic analyses for SCE are the gold standard method for the diagnosis of BS. Cytogenetic analysis is considered a fast and low-cost method to confirm the diagnosis of BS, and this methodology can be implemented in routine and diagnostic laboratories. Regardless, other molecular studies will also be conducted in living patients.

## Conclusions

BS is a rare, autosomal recessive, chromosomal instability disorder with remarkably increased risk for developing neoplasias at a young age. The neoplasias represent the main cause of death among affected individuals. Awareness of the high frequency of solid tumors among patients affected by this disorder must be raised because these individuals may benefit from individualized screening for solid tumors, including WT.

## Consent

Written informed consent was obtained from the patients’ legal guardians for the publication of this manuscript and the accompanying images. Copies of the written consents are available for review by the Editor-in-Chief of this journal.

## Competing interests

The authors’ declare that they have no competing interests.

## Authors’ contributions

MBM performed cytogenetic studies and wrote the paper. CRDCQ and CAK collected the patients’ clinical data. ACZT, GMNF and EAZ performed the cytogenetic studies. LDK and CAK coordinated the study and helped draft the manuscript. All authors read and approved the final manuscript.
